# Multiscale Modeling for Application-Oriented Optimization of Resistive Random-Access Memory

**DOI:** 10.3390/ma12213461

**Published:** 2019-10-23

**Authors:** Paolo La Torraca, Francesco Maria Puglisi, Andrea Padovani, Luca Larcher

**Affiliations:** 1 Dipartimento di Scienze e Metodi dell’Ingegneria, Università di Modena e Reggio Emilia, Via Amendola 2, 42122 Reggio Emilia, Italy; Luca_Larcher@amat.com; 2 Dipartimento di Ingegneria “Enzo Ferrari”, Università di Modena e Reggio Emilia, Via P. Vivarelli 10/1, 41125 Modena, Italy; francescomaria.puglisi@unimore.it; 3 Applied Materials, Via Sicilia 32, 42122 Reggio Emilia, Italy; Andrea_Padovani@amat.com

**Keywords:** AI, neuromorphic computing, multiscale modeling, memristor, optimization, RRAM, simulation

## Abstract

Memristor-based neuromorphic systems have been proposed as a promising alternative to von Neumann computing architectures, which are currently challenged by the ever-increasing computational power required by modern artificial intelligence (AI) algorithms. The design and optimization of memristive devices for specific AI applications is thus of paramount importance, but still extremely complex, as many different physical mechanisms and their interactions have to be accounted for, which are, in many cases, not fully understood. The high complexity of the physical mechanisms involved and their partial comprehension are currently hampering the development of memristive devices and preventing their optimization. In this work, we tackle the application-oriented optimization of Resistive Random-Access Memory (RRAM) devices using a multiscale modeling platform. The considered platform includes all the involved physical mechanisms (i.e., charge transport and trapping, and ion generation, diffusion, and recombination) and accounts for the 3D electric and temperature field in the device. Thanks to its multiscale nature, the modeling platform allows RRAM devices to be simulated and the microscopic physical mechanisms involved to be investigated, the device performance to be connected to the material’s microscopic properties and geometries, the device electrical characteristics to be predicted, the effect of the forming conditions (i.e., temperature, compliance current, and voltage stress) on the device’s performance and variability to be evaluated, the analog resistance switching to be optimized, and the device’s reliability and failure causes to be investigated. The discussion of the presented simulation results provides useful insights for supporting the application-oriented optimization of RRAM technology according to specific AI applications, for the implementation of either non-volatile memories, deep neural networks, or spiking neural networks.

## 1. Introduction

Artificial Neural Networks (ANNs) are possibly the most prominent computational model used in modern artificial intelligence (AI) applications. The computational scheme of ANN, loosely based on biological neural networks, comprises a collection of interconnected processing elements, referred to as artificial neurons, often organized in layers [1,2].

An ever-increasing effort has been devoted to the experimentation of different ANN architectures, which has led to the development of ANNs constituted by an extremely high number of neurons and layers [1,2], known as deep neural networks (DNNs). Thanks to their high flexibility and application-agnostic nature, DNNs have proved extremely effective in a variety of applications, such as image recognition [3,4] and speech recognition [5], easily outperforming other ANN architectures and machine learning techniques (i.e., Support Vector Machines, K-Nearest Neighbors).

However, the exceptional performance shown by the most advanced DNNs requires a heavy investment in terms of energy and hardware resources, during both the training of the network and the inference [6,7,8]. In fact, in most practical applications, DNNs are implemented in software and executed on von Neumann computer architectures that have to provide (i) sufficient computational power for fast training and inference, and (ii) a sufficiently large and fast memory for storing all the artificial neuron weights and any partial result. Nonetheless, the energy efficiency of training and inference must also be considered.

In order to achieve fast and efficient DNNs, computation has been progressively shifted from central processing units (CPUs) to optimized coprocessors, referred to as AI accelerators or Neural Processing Units (NPUs), that enable high parallelization by exploiting the DNN layered topology. Graphical Processing Units (GPUs) have readily been used as NPUs for their inherent capability to conduct efficient vector and matrix operations [9], followed by the development of the first Tensor Processing Units (TPUs), capable of efficient tensor operations and better data reuse [10]. Application-specific integrated circuits (ASICs) have recently been developed as special-purpose NPUs, co-designed with the desired DNN, exchanging a low flexibility for a higher energy efficiency, an optimized memory size and use, and a low area occupation [11,12,13,14].

Despite their steady improvements, even ASIC NPUs are now facing technological limits (i.e., the imminent end of Moore’s law and the already broken Dennard scaling) and the intrinsic limits of the von Neumann architecture, mainly the so-called von Neumann bottleneck (i.e., the limited data transfer speed between the processor and memory, as well as the energy required for the data transfer itself). This is especially true in applications with strict volume, weight, and power constraints, such as automotive, battery-powered vehicles (i.e., drones); mobile devices; and Internet of Things (IoT) devices. In this context, memristor-based neuromorphic systems can potentially overcome the limitations posed by von Neumann architectures, not only to DNNs, but to AI in general.

Memristors are electronic passive devices characterized by a pinched hysteresis I-V curve, thus exhibiting a time-varying and non-volatile electrical resistance [15,16]. Ideally, the conductivity of a memristor can be arbitrarily modified by applying a proper electrical stimulus, indefinitely retaining the resistance state in the absence of external stimuli.

The peculiar memristor characteristics, combined with their extremely small size, have made them very appealing for the development of new non-volatile memories (NVMs), characterized by a low power, high density, and possibly multilevel data storage [17,18].

Even more importantly for AI applications, memristive crossbar arrays have been proposed as DNN accelerators, natively implementing an in-memory (typically analog) vector-matrix multiplication [18,19,20], which is the fundamental operation for the computation of a layer output in DNNs. By storing the weights of a DNN layer in the memristive array as conductance values, matrix-vector multiplication can be executed at once by only exploiting Ohm’s and Kirchhoff’s laws. The computation of a single DNN layer requires a single execution step and, moreover, it is performed at the data location, preventing the massive data transfer of weight values between the memory and processor required in von Neumann architectures.

Memristors are also finding applications in more biologically-plausible neural networks, such as Spiking Neural Networks (SNNs), in which the computation is performed by mimicking the actual operation of biological neurons (i.e., spatio-temporal coding of the information, synaptic plasticity, cell membrane V-I relationship, generation of action potentials, and intrinsically stochastic behavior) [18,19,21]. Different memristor-based artificial synapse implementations have been proposed, and have successfully exhibited both deterministic and stochastic spike-time-dependent plasticity (STDP) [21,22]. Their application to SNNs has highlighted the potential for supervised and unsupervised learning, and adaptation to input stimuli. A recent analysis on the Hodgkin–Huxley neuron model [23], known for being the most biologically-sound model of the neuron action potential, suggested that memristors are key components for its implementation [24]. Moreover, the intrinsic stochasticity of real memristors can be exploited for mimicking the probabilistic and noisy behavior of biological neurons [22], enabling more biologically-plausible artificial neuron implementations and overcoming the limits of the strictly deterministic CMOS implementations. Memristor-based SNNs thus have a huge potential, providing a new architectural and computational paradigm approaching a brain-like computation and circumventing the von Neumann bottleneck at once.

Since the fabrication of the first TiO_2_ memristor in 2008 [25], many memristor technologies have been proposed [19,26]. In Resistive Random-Access Memories (RRAMs), the switching is induced by the formation of a conductive path in a dielectric material, controlled by an ion-based mechanism [27,28,29,30,31]. In Phase-Change Memories (PCM), the modulation of a chalcogenide material phase (i.e., amorphous and crystalline) through localized Joule heating allows the resistance switching [32,33,34]. In a Ferroelectric Tunnel Junction (FTJ), the tunneling electroresistance of a ferroelectric material is modulated by setting its internal polarization [35,36,37]. In a Magnetic Tunnel Junction (MTJ), the tunneling electroresistance of a thin insulator enclosed between two ferromagnetic layers is modulated by their magnetic polarization [38].

Although the proposed memristor technologies are characterized by a simple structure and are easy to fabricate, the physical principles underlying their analog resistance switching and, in general, the implications of the atomic material properties (i.e., defects, phase, morphology) on the electrical performances, are extremely complex and are still not comprehensively understood. This lack of knowledge is currently hampering the development of memristor-based systems.

In fact, all the memristor applications previously discussed ask for devices with different performance metrics, as summarized in Table 1. Memristors must thus be appropriately chosen, designed, and optimized to satisfy the performance requirements for each specific application. This, in turn, requires a deep knowledge of the physical mechanisms responsible for the resistance switching phenomena, their interplay, and how they are affected by the device materials and geometry. However, the partial comprehension of the physical mechanisms underlying memristor operation prevents the application-oriented design and tuning of the devices.

Physical multiscale modeling and simulation provide a powerful tool for investigating the physical mechanisms responsible for analog switching in memristors and to highlight the effects of the material properties (including defects) on the device performance. This information can then be used to further develop memristive technology, optimizing the properties of the devices (i.e., geometry and materials) to match the specifications required for the desired application (i.e., electric properties, data retention, variability, and noise), and strongly reducing its time-to-market.

In this paper, we use a multiscale modeling platform to simulate RRAM devices and investigate the physical mechanisms underlying their operation. The simulations are designed to highlight the effects of the device geometry, materials, forming conditions (i.e., temperature, current compliance, voltage stress mode), and programming. The discussion on the simulation results shows the relevance of the obtained insights for the different AI applications (i.e., non-volatile memories, deep neural networks, or spiking neural networks) and provides useful design principles for RRAM application-oriented design.

## 2. Materials and Methods

As highlighted in Section 1, the design of memristive devices requires complete knowledge of all the involved resistance switching phenomena, including the effects of the device geometry and materials.

In RRAMs, the fundamental mechanisms include (i) the interaction between the electronic and ionic transport, (ii) the effects induced by the applied electric field, and (iii) the influence of the microscopic material properties [41].

Due to the complexity of those mechanisms, exacerbated by their interplay, the physical modeling of RRAMs presented in this work is extremely advantageous, as it easily allows the following: (i) prediction of the device performance (i.e., switching time, endurance, and retention) from the material properties (which is one of the key novel aspects of this work, which allows the materials and the process conditions to be directly screened when targeting specific applications); (ii) investigation of the trade-off between the device scaling and variability; (iii) evaluation of the process effects on the device and its materials through the interpretation of electrical characterization data; and (iv) co-design of the device materials and geometries for satisfying the specific application requirements (see Table 1).

In this section, we first review the physical mechanism underlying the RRAM operation, and then present a multiscale modeling platform that includes all the presented effects and mechanisms.

### 2.1. RRAM Devices

In RRAMs, the analog resistance switching is controlled by a reversible, voltage-driven, and ion-based mechanism that allows the geometry of a conductive path within a dielectric layer to be modulated. Different switching mechanisms have been proposed for the implementation of RRAM [26,42,43], with each one requiring the comprehensive knowledge of different physical processes for being thoroughly understood.

In CBRAMs [44,45,46], a conductive filament (CF) is created (and dissolved) in a solid electrolyte by means of redox reactions. By applying a positive voltage to the “active” electrode (the anode), it releases metallic cations into the electrolyte that migrate towards the “inert” electrode (the cathode) pushed by the electric field. Once they reach the cathode, the cations are reduced, contributing to the formation of the CF and eventually connecting the two electrodes. Through reversing the redox process by applying a negative voltage to the anode, the CF is progressively dissolved.

Optimizing CBRAMs requires an understanding of the kinetics and interplay between the redox processes and the transport of metallic cations in solid electrolytes (i.e., drift and diffusion). Moreover, all the effects related to the material interactions and geometry must be considered.

**Figure 1:**
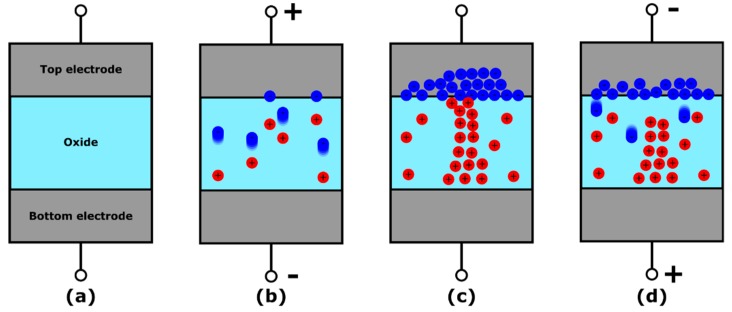
Structure and operation of a filamentary OxRAM device. (**a**) Device in pristine conditions. (**b**) Forming operation: a positive voltage is applied to the top electrode, generating oxygen ion/vacancy pairs. The ions (blue spheres) migrate under the effect of the electric field, leaving the oxygen vacancies (red spheres) behind. (**c**) Formed device with a conductive filament (CF) made of oxygen vacancies connecting the two electrodes. (**d**) Reset operation: a negative voltage is applied to the top electrode, bringing the oxygen ions near to the oxygen vacancies. The resulting recombination leads to partial dissolution of the CF and the formation of a dielectric barrier.

In OxRAMs [47,48,49,50,51], a conductive path constructed of oxygen vacancies is formed in a high-k oxide layer (e.g., TiO_2_, HfO_2_, TaO_5_), breaking the oxide atomic bonds (Figure 1a).

When applying a voltage to the oxide layer (Figure 1b), its lattice bonds are stretched. With a sufficiently high voltage, the bonds eventually break, generating a negatively charged oxygen ion and a positively charged oxygen vacancy (Frenkel pair). If no recombination of the pair occurs, the oxygen ion and vacancies migrate in opposite directions under the action of the electric field. However, due to their relatively high diffusion energy barrier [52], the oxygen vacancies experience little to no motion, locally increasing the electrical conduction and power dissipation in the material. The resulting temperature increment supports the creation of new oxygen ions/vacancies, leading to the rapid formation of a highly conductive path of oxygen vacancies between the two electrodes (Figure 1c). The conductive path can be broken by recombining the oxygen vacancies with the oxygen ions, i.e., bringing the oxygen ions near the oxygen vacancies by applying a negative voltage to the oxide layer (Figure 1d).

The analog resistance switching in OxRAMs relies on the modulation of a conductive path created during a forming process, consisting of a current-controlled breakdown of the dielectric. After the formation, only a thin portion of the formed conductive path is affected by the oxygen ion/vacancies generation and recombination processes. The conductance switching thus requires precise control of the geometrical properties of a thin insulating barrier.

Depending on the forming conditions (i.e., temperature, current compliance, voltage stress mode), the conductive path can assume either a uniform or filamentary shape. In the former [51], the dielectric conduction is uniformly modulated across its section and the resistance is thus controlled through the insulating barrier thickness only. In the latter [47,48,49,50], a CF is formed within the dielectric, enabling control of the resistance by both the barrier thickness and the CF diameter.

The design of OxRAMs requires an understanding of the complex processes of oxygen ions/vacancies generation, diffusion, and recombination under the action of an external electric field; their interplay with the charge transport in dielectrics; and the effects of the material properties and geometries on all those processes.

In this work, we focus on the multiscale simulation of OxRAM devices, and investigate the interplay between the physical mechanisms involved and their impact on the device performance, reliability, and variability. Specifically, we mainly consider OxRAM devices with a one-oxide-layer stack made of TiN/HfO_x_/TiO_y_/TiN (where TiO_y_ is a parasitic layer). For comparison, we also consider a two-oxide-layer stack made of TiN/Ta_2_O_x_/TiO_y_/TiN, showing the different properties enabled by such structural and material combination.

### 2.2. Multiscale Modeling of RRAMs

**Figure 2:**
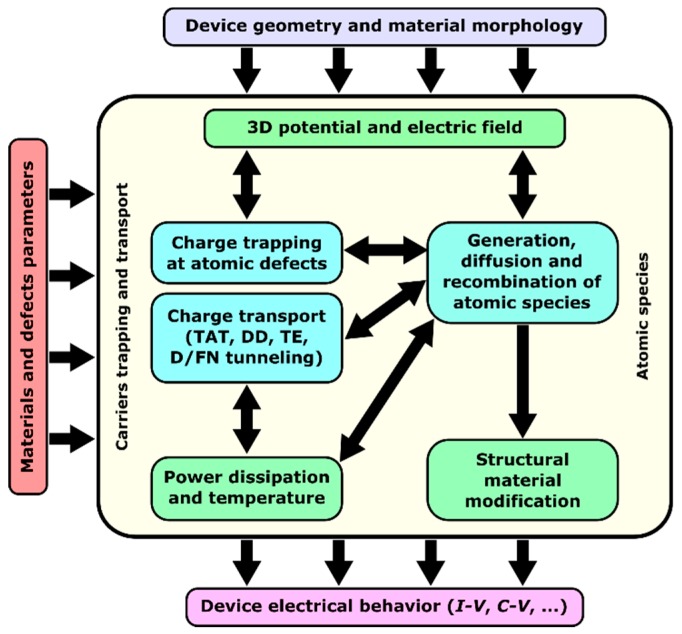
Multiscale modeling platform for RRAM devices. The device-level simulation engine includes three main modules: one for the simulation of charge transport; one for charge trapping; and one for the simulation of atomic species generation, diffusion, and recombination. The simulation requires the material parameters, calculated using ab-initio methods, and a definition of the device geometry. The modeling platform considers the 3D potential and electric field and how they are affected by localized trapped charge and power dissipation at defects. The results are the various characteristic curves describing the device electrical response.

The multiscale modeling platform sketched in Figure 2 allows the RRAM devices to be thoroughly investigated. Starting from the key material properties, calculated using ab-initio methods [53,54,55], and the other device-specific properties (e.g., geometry and materials), the platform models the electrical device response considering all the complex physical mechanisms involved in the different RRAM operations, while accounting for all the resulting changes in the 3D electric and temperature fields, and in the material structure.

The multiscale modeling platform comprises three main modules, addressing charge transport, charge trapping, and the generation/diffusion/recombination of atomic species (i.e., oxygen vacancies and interstitial ions), respectively.

For a comprehensive simulation of charge transport, many mechanisms are considered, including trap-assisted tunneling (TAT), thermo-ionic emission (TE), the Poole–Frenkel effect (PF), and Drift-Diffusion (DD) through conduction and valence bands, and through sub-bands originating from metal-rich regions formed within the oxide. Interestingly, in most RRAM devices, the TAT and DD phenomena dominate the charge transport dynamics, the first at low defect densities (i.e., the conduction in the oxide is mainly defect-assisted, typical of binary and ternary oxides [56,57,58,59,60,61,62]), and the latter for sufficiently high defect densities (i.e., a conductive path is present within the oxide). The TE and the PF phenomena are typically negligible in materials with a medium/high bandgap, such as transition metal oxides [63].

The TAT conduction is described using a multiphonon TAT model, inherently considering the electron-phonon coupling oxides by accounting for the atomic lattice rearrangement in the vicinity of the defect due to the presence of a trapped charge [56,57,58,64]. The TAT transport considered is fully described in [65]. This model requires knowledge of the relaxation energy, E_REL_, associated with the atomic lattice relaxation process; the defect thermal ionization energy, E_T_; and the defect density in the material, N_T_. Both E_REL_ and E_T_ are calculated by means of ab initio methods (i.e., Density Functional Theory and Molecular Dynamics simulations). The DD conduction is best described by adopting the Landauer approach [66], which accounts for delocalized electron flow in the conductive path.

The defect-assisted charge transport naturally results in localized power dissipation at the defect sites, affecting the temperature distribution in the device. The power dissipation is self-consistently computed across the entire device volume by including the charge carrier’s energy released at both the defects (at every charge trapping event) and the lattice (due to inelastic scattering mechanisms, i.e., optical and acoustic phonons). The temperature distribution in the device volume is calculated from the power dissipation by solving the Fourier’s Law for heat conduction.

The charge transport dynamics are strongly coupled to the different phenomena related to atomic species in the oxide (i.e., generation, diffusion, recombination). Therefore, they must be consistently calculated to effectively model the structural material modifications occurring during RRAM operations (i.e., forming, setting, and resetting).

The generation of atomic species is described by a thermochemical bond breakage model [67], consisting of compact effective-energy formulas accounting for the microscopic material properties and the two main generation mechanisms: (i) the breakage of atomic bonds, enhanced by the local electric and temperature field profiles and locally favored by the possible presence of precursors [68] and other defects, and (ii) the redox reactions that occur at the interfaces, as well-favored by the local electric and temperature fields. The resulting generation rate is

(1) $$G\left(x,y,z\right)=G_{0,G}\exp\left[-\frac{E_{A,G}\;-\;b\cdot F\left(x,y,z\right)}{k_{B}T\left(x,y,z\right)}\right],$$

where $b=p_{0}\frac{2+k}{3}$ is the bond polarization factor related to the molecular dipole moment, $p_{0}$ is the bond breakage activation energy, k is the material dielectric constant, $G_{0,G}$ is the effective bond vibration frequency, $E_{A,G}$ is the bond breakage activation energy, F(x,y,z) is the 3-D electric field, $k_{B}$ is the Boltzmann’s constant, and T(x,y,z,) is the 3-D temperature field. The material-related parameters (i.e., the effective bond vibration frequency $G_{0,G}$, the molecular bond polarizability $p_{0}$, and the bond breakage activation energy $E_{A,G}$) are calculated using ab-initio methods [54,69].

The transport of atomic species is dominated by a DD mechanism, driven by the electric field and strongly accelerated by the local temperature, described by the equation

(2) $$G_{D}\left(x,y,z\right)=G_{0,D}\exp\left[-\frac{E_{A,D}\;-\gamma \cdot E\left(x,y,z\right)}{k_{B}T\left(x,y,z\right)}\right],$$

where $G_{0,D}$ is the effective bond vibration frequency, $E_{A,D}$ is the diffusion activation energy, γ is the field acceleration factor, F(x,y,z) is the 3-D electric field, $k_{B}$ is the Boltzmann’s constant, and T(x,y,z,) is the 3-D temperature field.

However, both the electric and temperature fields are in turn affected by the presence of atomic species, implying a strong and complex coupling between the transport mechanism and the fields in the device. For correctly modeling the DD transport, the internal device conditions (e.g., current, trapped charge distribution, electric and temperature fields) are updated every time an individual defect is generated, recombined, or moved.

The stochastic nature of the mechanisms involved in RRAM is successfully accounted for by using a kinetic Monte Carlo approach, which allows a consideration of phenomena like the intrinsic variability of the forming, set, and reset processes, and the occurrence of Random Telegraph Noise [70,71,72,73,74], together with their dependence on the material properties and geometry, providing insights for their optimization.

The presented modeling platform successfully reproduces the RRAM electrical responses to arbitrary voltage and current inputs, allowing for the extraction of various characteristic curves of the device (I-V, C-V, and G-V). The parameters used in all the simulations are reported in Table 2.

## 3. Results

The multiscale modeling platform presented in Section 2 combines all the most relevant physical mechanisms involved in RRAM operation, directly connecting the electrical performance of RRAM devices to their geometries and to the microscopic properties of the employed materials. Noticeably, thanks to the multiscale nature of the modeling platform, it can be used to gain insights into RRAM devices at different levels.

At the physical level, the modeling platform can be used to investigate the included physical mechanisms and the effects of their interplay on the generation of the conductive path. At the device level, it allows the whole conductance switching cycle (i.e., forming, set, and reset operations) to be simulated, and can therefore be used to predict the performance of RRAM devices. Moreover, its inherently stochastic implementation allows the device variability and reliability (retention and endurance) properties to be effectively investigated. Multiscale modeling thus provides a powerful tool for accelerating the further development and optimization of RRAM technology, focusing on the specific application (i.e., NVM, ANN, or SNN).

In this section, we use the presented multiscale modeling platform to perform multiscale simulations of RRAM devices. The different simulations are specifically designed to highlight the effects of the device geometry, materials, forming conditions (i.e., temperature, current compliance, voltage stress mode), and programming. The parameters used for the simulations are summarized in Table 2.

### 3.1. Conductance Switching Cycle

The presented multiscale modeling platform allows the whole conductance switching cycle to be simulated. Starting from a pristine device with specified geometries and materials, it is possible to investigate the device formation and the following conductance switching operations (SET and RESET) under different conditions. The electric and temperature fields and the location of atomic species in the device can also be monitored during simulations to gain insights into the involved switching mechanisms and their interplay.

**Figure 3:**
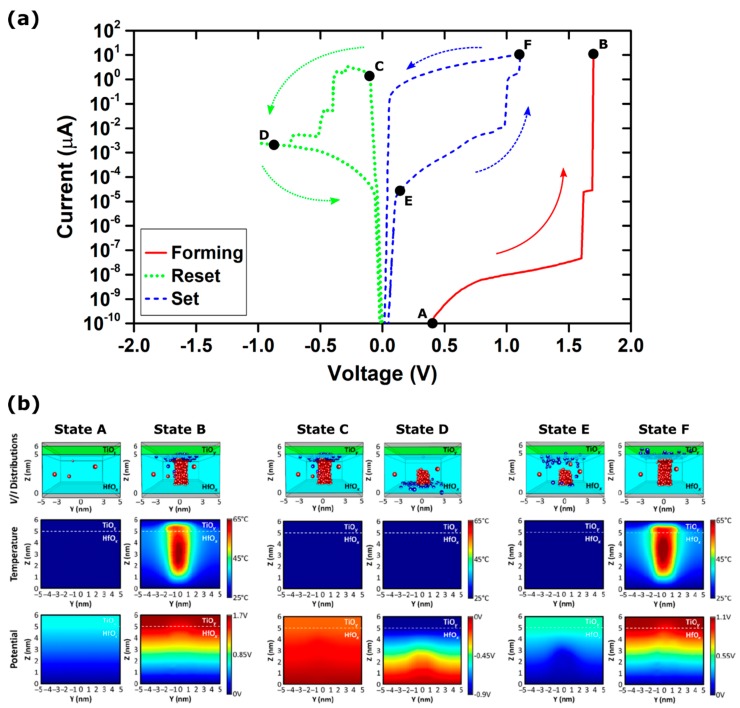
Simulation results of a TiN/5 nm HfO_x_/TiO_y_/TiN stack RRAM device: (**a**) Current–Voltage characteristic during forming (solid red), reset (dotted green), and set (dashed blue) operations with a compliance current of 10 µA; (**b**) oxygen vacancy (red spheres) and ion (blue spheres) distribution, temperature profile, and potential profile of the simulated device at different operation stages (labeled A, B, C, D, E, and F in Figure 3a).

As an example, Figure 3a shows the I-V characteristic curves of a simulated device made of a TiN/5 nm HfO_x_/TiO_y_/TiN stack RRAM device, including the forming (solid red), the reset (dotted green), and set (dashed blue) operations. The simulations are performed by applying ramped voltages and a compliance current of 10 µA.

In the pristine state (state A), the conduction is dominated by the TAT through the relatively few preexisting defects in the material (i.e., oxygen vacancies accumulated at grain boundaries), which determines the very high initial resistance of the device. At a low voltage (V < V_FORM_ = 1.7 V in Figure 3), both the electric field and the power dissipation are too small to result in strong localized defect generation, leading to a very modest and uniform generation of defects across the whole volume of the device. Once the applied voltage exceeds V_FORM_, the oxide bonds start to break under the effect of the increasing electric field, creating a significant number of atomic defects. Due to the different diffusion energy barriers, the oxygen ions generated drift towards the top electrode under the effect of the electric field, while the oxygen vacancies mostly remain in place. Noticeably, the oxygen ions accumulate at the TiO_x_ layer, creating the so-called “oxygen reservoir”. The newly created oxygen vacancies support the TAT, locally increasing the current flow, power dissipation, and temperature, in turn assisting with the generation of new defects. A thermally-driven positive feedback process is thus established, leading to the rapid formation of a CF. After the formation (state B), the device is in a low resistance state. The conduction is dominated by DD in the vacancy-rich regions constituting the CF, while the oxygen ion counterparts are gathered at the top electrode.

The reset operation is simulated by the device configuration resulting from the described forming operation. This approach is advantageous since, in comparison to other approaches proposed in the literature [66,75], no a-priori assumptions of the CF structure and characteristics are required. Upon the application of a negative voltage ramp (state C), the oxygen ions gather in the oxygen reservoir during the forming drift towards the bottom electrode under the effect of the electric field. During their motion, the oxygen ions can recombine with the oxygen vacancy defects in the oxide, leading to the progressive formation of a thin dielectric barrier within the CF, causing resistive switching to the high resistance state (state D). Interestingly, this process is not associated with a large temperature increment in the device, suggesting that it is mostly driven by the electric field.

Lastly, the set operation is simulated by the device configuration obtained at the end of the reset operation. Upon the application of a positive voltage ramp (state E), the device experiences a process similar to that of forming, but confined to the thin dielectric barrier created during the reset process. Since the applied voltage drops almost completely across the thin dielectric barrier, the electric field required for the breaking of oxide bonds and the generation of new defects is easily exceeded at relatively low voltages (V < V_FORM_). The restoration of the CF is thus initiated at lower voltages compared to the forming process. The same thermally-driven positive feedback described for the forming process is established, resulting in a quick restoration of the CF and the switch to a low resistance state (state F).

### 3.2. Effects of Forming Conditions

The presented multiscale modeling platform allows the effects of the forming condition (i.e., temperature, current compliance, voltage stress mode) on the performance and properties exhibited by the device after the forming process to be investigated.

The beneficial effects of a high-temperature forming process have been thoroughly reported in the literature, and have been associated with lower forming voltages and variability of the low resistance state, while improving the stability and reliability of the device [76,77,78]. The external temperature affects the forming process assisting the defect generation and promoting the oxygen ion diffusion in the device, leading to a lower density of oxygen ions near the conductive path after the forming process. The subsequent oxygen ion/vacancy recombination is strongly reduced, resulting in a higher stability and lower variability of the conductive path.

**Figure 4:**
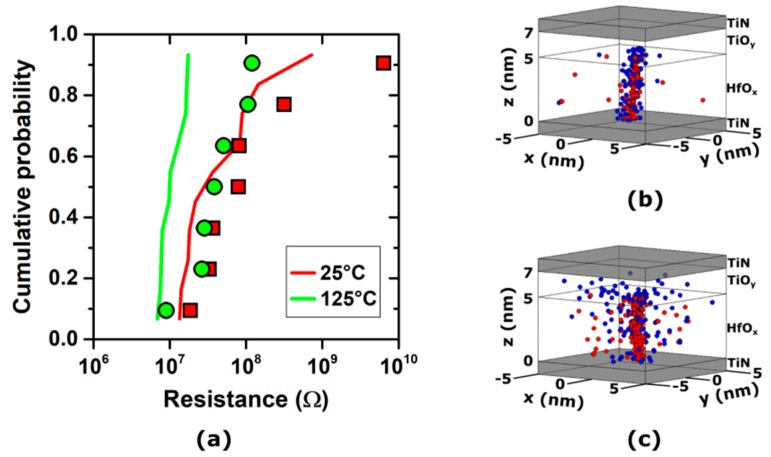
(**a**) Experimental (symbols) and simulated (lines) cumulative distributions of TiN/5 nm HfO_x_/TiO_y_/TiN RRAM stacks’ low state resistances after forming at 25 and 125 °C. (**b**) Resulting oxygen ions/vacancies distribution in the device after forming at 25 °C. (**c**) Resulting oxygen ions/vacancies distribution in the device after forming at 125 °C. The forming process was performed using a ramped voltage and a 1 µA compliance current.

This is evidenced in Figure 4a, which shows the low state resistance distribution exhibited by TiN/5 nm HfO_x_/TiO_y_/TiN RRAM stacks after the forming process at two different external temperatures (i.e., 25 and 125 °C), using a ramped voltage and a 1 µA compliance current. In accordance with the literature [76], the experimental data (marked by the symbols in Figure 4a) show that the higher forming temperature leads to a tighter resistance distribution.

The effects of the forming temperature on the oxygen ions/vacancies generation and diffusion can be effectively investigated using the presented multiscale modeling platform. The simulations of the forming processes (shown by the lines in Figure 4), whilst not perfectly matching the measured samples, accurately reproduce the trend and order of magnitude of the experimental data, showing a tighter resistance distribution in the higher temperature forming case. The discrepancy between the experimental results and those of the simulation can be ascribed to several effects not considered in these simulations, mainly, the oxide thickness and area variations due to the fabrication process tolerances, and process-dependent interface effects between the oxide and the electrodes.

The microscopic differences caused by the higher forming temperature can be better appreciated in Figure 4b,c, which shows the simulated oxygen ions/vacancies distribution in the device after the forming process. At a low temperature (25 °C), the conductive path (in this case, a CF) made of oxygen vacancies is tightly surrounded by oxygen ions affecting its resistance and stability. Conversely, at a high temperature (125 °C), the oxygen ions are scattered in the device volume, affecting the CF to a lesser extent and leading to a tighter resistance distribution.

The platform can therefore be used to explore the best strategies to control the device-to-device variability of RRAMs for specific applications: for instance, high-temperature forming could reduce the variability for high-accuracy DNNs, while precisely controlling the temperature during the forming process could be used to obtain the desired variability level, optimizing the performance of stochastic learning networks.

**Figure 5:**
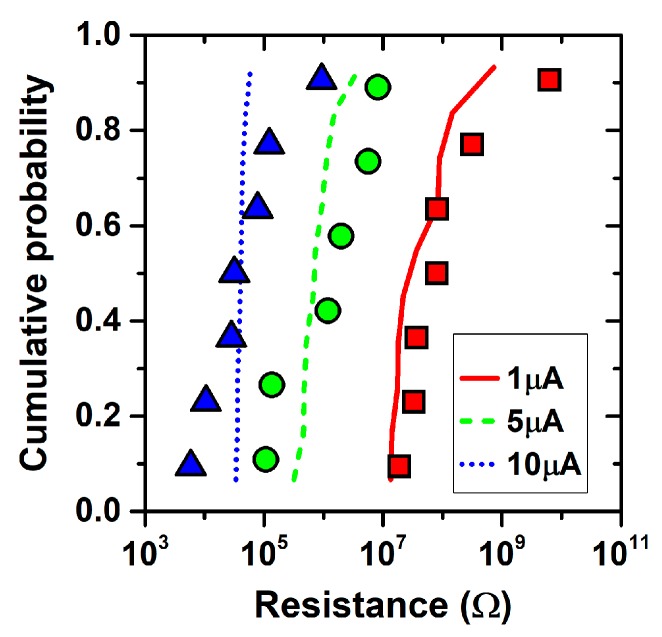
Experimental (symbols) and simulated (lines) cumulative distributions of TiN/5 nm HfO_x_/TiO_y_/TiN RRAM stacks’ low state resistances after forming at 1, 5, and 10 µA. The forming process was performed using a ramped voltage and 25 °C external temperature.

The forming compliance current has a very strong impact on the morphology of the conductive path as it allows the defect generation processes in the device to be controlled by limiting the maximum current flow and power dissipation in the device. For example, in filamentary RRAMs, it has been observed that the magnitude of the compliance current allows the diameter of the CF to be controlled [57]. Moreover, it also greatly affects the low resistance state magnitude and variability: a sufficiently high compliance current allows the formation of a dense population of defects, forming low-resistance CFs with a similar morphology, while a low compliance current leads to a sparse population of defects, forming weak CF characterized by a larger variability. This is evidenced in Figure 5, which shows the low state resistance distribution exhibited by TiN/5 nm HfO_x_/TiO_y_/TiN RRAM stacks after the forming process at three different compliance currents (i.e., 1, 5, and 10 µA), using a ramped voltage and an external temperature of 25 °C.

The effects of the compliance current on the defect generation processes and the resulting device resistance magnitude and variability can be effectively investigated using the presented multiscale modeling platform. The simulations of the forming processes, shown in Figure 5, reproduce the trend found in the experiments, highlighting that the lower variability of the low resistance state is in fact related to the higher density of oxygen vacancies generated at higher compliance currents. The platform confirms its effectiveness for investigating the best strategies to control the device-to-device variability for specific applications, as previously mentioned.

In filamentary RRAM, the morphology of the CF is also strongly affected by the forming voltage stress mode (i.e., the time-varying profile of the forming voltage), as it determines different distributions of oxygen ions and vacancies in the device volume. Evaluating the effect of a specific voltage stress mode is highly desirable, since it allows the best strategy for a specific application to be identified. However, this task is extremely complex, as it requires the interplay between the (possibly) time-varying forming voltage, the defect generation processes driving the growth of the CF, and the oxygen ions diffusion to be considered. The presented multiscale modeling platform provides a powerful tool for such investigation.

**Figure 6:**
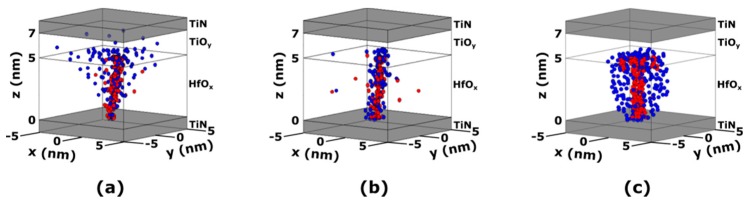
Simulated distributions of the oxygen ions (blue) and vacancies (red) in TiN/5 nm HfO_x_/TiO_y_/TiN RRAM stacks after forming with a (**a**) constant voltage, (**b**) ramped voltage, and (**c**) pulsed voltage. The forming process was performed using a 1 µA compliance current and 25 °C external temperature.

Figure 6 shows the simulation of the oxygen ions/vacancies distribution in TiN/5 nm HfO_x_/TiO_y_/TiN RRAM stacks after the forming process using three different voltage stress modes (i.e., constant voltage, ramped voltage, and pulsed voltage), a compliance current of 1 µA, and an external temperature of 25 °C.

Noticeably, the oxygen ions distribution is significantly affected by the voltage stress mode. At constant voltage forming, the oxygen ions are mostly accumulated near the top electrode, while ramped and pulsed voltage forming results in a more uniform distribution. The pulsed voltage forming, however, leads to a higher radial diffusion of the oxygen ions compared to the constant voltage forming.

Comparing the oxygen ions and vacancies distribution shown in Figure 4 and Figure 6, it can be noticed that the distribution corresponding to a low-temperature forming process (Figure 4b) is similar to the one of a ramped voltage forming process (Figure 6b). Conversely, the distribution corresponding to a high-temperature forming process (Figure 4c) is closer to that of a pulsed voltage forming process (Figure 6c). The similarity of the oxygen ions and vacancies suggests a similar behavior of the device. This is confirmed by experimental results [79], showing that pulsed voltage forming is associated with a tighter resistance distribution with respect to constant or ramped voltage forming.

The voltage stress mode can thus be exploited for optimization of the oxygen ions distribution in the formed device for achieving a tighter resistance distribution, similar to the previously discussed forming temperature. The platform can support such optimization, allowing for the fine tuning of RRAM device-to-device variability according to the specific application requirements.

### 3.3. Analog Resistance Switching Optimization

As previously discussed, the analog resistance switching in RRAM devices is driven by two different mechanisms. The switching from a high resistance state to a low resistance state is dominated by thermal positive feedback triggered by an electric field, leading to a mostly uncontrolled generation of oxygen vacancies and resulting in the abrupt formation of a conductive path. Conversely, the switching from a low resistance state to a high resistance state is determined by the recombination of oxygen ions/vacancies driven by the electric field, resulting in the new formation of a high-resistance dielectric barrier. The dielectric barrier formation, being unassisted by the temperature, is typically slower than the formation of the conductive path.

The differences between the two switching mechanisms cause strong asymmetry in the device characteristics, as evidenced in Figure 3, which has been recognized as detrimental for many applications (i.e., ANNs and SNNs). Moreover, the extremely fast formation of the conductive path prevents the fine control of analog resistance switching, potentially limiting both the number of distinguishable resistance levels (thus the number of bits per cell in NVM) and the device linearity.

A well-established method for mitigating the non-idealities exhibited by RRAM devices is pulsed programming, i.e., controlling the resistance switching by applying a sequence of voltage pulses to the device. Ideally, each set (reset) voltage pulse should decrease (increase) the device resistance by a small amount, according to the device’s electrical characteristics, enabling fine tuning of the resistance [80].

In the simplest approach, the applied pulses are all identical in amplitude and width (with opposite signs in set and reset operations), allowing for better control of the conductive path formation. However, this is often not sufficient for the full compensation of RRAM non-idealities, requiring sequences of non-identical pulses and possibly a combination of positive and negative pulses for each set or reset operation [80]. Fine tuning of the pulse sequence shape, amplitude, and width is thus of paramount importance for exploiting the full potential of RRAM devices.

A combined design of the device geometries and materials can be beneficial for controlling the RRAM non-idealities and achieving linear resistance analog switching without using complex voltage pulse schemes [81]. A simple solution consists of using a two-layer RRAM stack made of two different dielectric materials, comprising a thin (~1–2 nm) low-k (LK) material and a thicker high-k (HK) material. The resulting distribution of the electric field across the device allows the switching mechanism to be controlled and gradual modulation of the device resistance to be produced. In such a configuration, the structural material changes responsible for the resistance switching (i.e., the conductive path and dielectric barrier formation) are confined to the LK layer, where the electric field is the highest, regardless of the layers’ thickness. The confinement of these phenomena in the LK layer is the key factor that leads to a gradual change of the electrical resistance in the device, which is crucial to achieving precise and linear analog switching. Moreover, this two-layer structure allows for better control of the oxygen ions diffusion. The low electric field in the HK layer hinders the diffusion of oxygen ions coming from the LK layer, leading to the formation of an ion reservoir at the LK–HK interface. Having the oxygen ions reservoir near the conductive path contributes to smoothening the whole switching process and results in gradual modulation of the electrical resistance, with obvious benefits for applications requiring a linear and gradual conductance change, e.g., DNNs.

The presented multiscale modeling platform allows the devices’ non-idealities and their effects on the device performance (i.e., symmetry, linearity, number of levels) to be evaluated. The retrieved information can then be exploited, supporting optimization of the device structure and materials, and the programming pulse sequence for achieving linear analog resistance switching.

**Figure 7:**
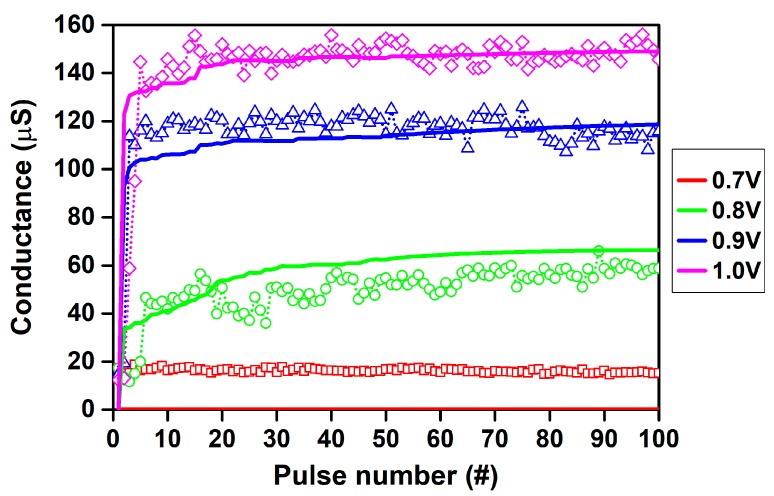
Experimental (symbols) and simulated (lines) conductance evolution of TiN/6 nm HfO_x_/TiO_y_/TiN RRAM stacks under the application of set pulse trains with a 1 ms width and variable amplitude: (red) 0.7 V; (green) 0.8 V; (blue) 0.9 V; (magenta) 1.0 V. Initial forming was performed with a 100 µA compliance current.

Starting from a one-layer device, Figure 7 shows the experimental and simulated conductance evolution of TiN/6 nm HfO_x_/TiO_y_/TiN RRAM stacks under the application of set pulse trains with an identical width (1 ms) and different voltage amplitudes (0.7, 0.8, 0.9, and 1 V). After each single set pulse, the conductance was read with a read pulse with 0.1 V amplitude and 1 ms width, which did not significantly influence the device resistance.

Noticeably, only pulse trains with amplitudes of 0.8 V and above induce a variation of the device conductance, mostly during the first few pulses (<5) of the sequence. The conductance quickly saturates, highlighting both the nonlinear characteristic of the device and the abrupt formation of the conductive path. These trends are accurately reproduced by the simulations. The fluctuation in conductance exhibited by the experimental results can be ascribed to the random nature of oxygen migration caused by the voltage pulses [82]. In fact, even after the conductance saturation, each pulse causes a small random motion of the oxygen ions in the RRAM stack, resulting in fluctuations of the conductance around a mean value.

Taking advantage of the RRAM stack’s nonlinear and saturating behavior, it is possible to associate a set conductance value with a specific pulse voltage. Further simulations could support the optimization of pulse amplitudes for the best discrimination of conductance levels.

**Figure 8:**
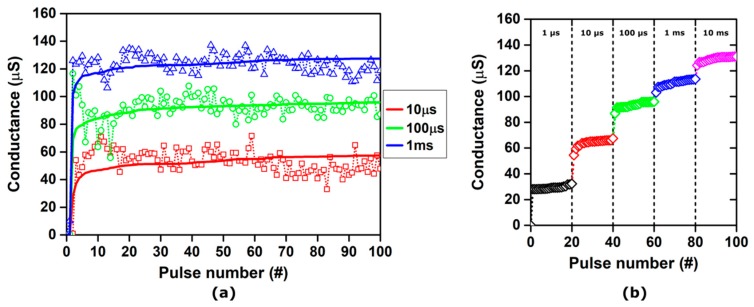
(**a**) Experimental (symbols) and simulated (lines) conductance evolution of TiN/6 nm HfO_x_/TiO_y_/TiN RRAM stacks under the application of set pulse trains with a 0.9 V amplitude and different widths: (red) 10 µS; (green) 100 µS; (blue) 1 ms. Initial forming was performed with a 100 µA compliance current. (**b**) Simulated conductance modulation obtained using set pulse trains with an increasing width: (black) 1 µS; (red) 10 µS; (green) 100 µS; (blue) 1 ms; (magenta) 10 ms.

Similar results are obtained by modulating the pulse width, as shown in Figure 8a, which depicts the experimental and simulated conductance evolution of the same device under the application of set pulse trains with an identical voltage amplitude (0.9 V) and different widths (10 µS, 100 µS, and 1 ms). As shown in Figure 8b, it is possible to take advantage of the nonlinear and saturating behavior exhibited by the considered RRAM stack to associate a set conductance value with a specific pulse width, which achieves six well-separated and recognizable resistance levels with an approximately linear characteristic. Finely tuning the programming pulse train for both set and reset processes allows a linear resistance update to be achieved for any specific RRAM device. The best combination of pulse amplitude, width, and sequence can be effectively investigated by the presented multiscale modeling platform, in order to optimize the synaptic behavior of the device. On the other hand, the platform could be useful for implementing design-circuit co-design strategies to enhance the device performance for multi-level memory applications, increasing the robustness and possibly reducing the bit error rate.

**Figure 9:**
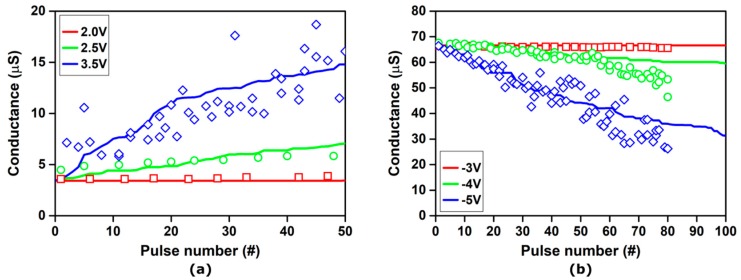
Experimental (symbols) and simulated (lines) conductance evolution of TiN/2 nm Ta_2_O_x_/35-nm TiO_y_/TiN RRAM stacks under the application of set pulse trains with a 100 µS width and variable amplitude. (**a**) Set operation: (red) 2 V; (green) 2.5 V; (blue) −3.5 V. (**b**) Reset operation: (red) −3 V; (green) −4 V; (blue) −5 V.

Using a two-layer RRAM stack made of a thin LK layer (i.e., Ta_2_O_5_) and a thick HK layer (i.e., TiO_2_), a linear conductance characteristic can be obtained. Figure 9 shows the experimental and simulated conductance evolution of TiN/2 nm Ta_2_O_x_/35-nm TiO_y_/TiN RRAM stacks during both set and reset operations, under the application of pulse trains with an identical width (100 µS) and different voltage amplitudes. After each single pulse, the conductance was read with a read pulse with a 0.1 V amplitude and 1 ms width, which did not significantly influence the device resistance.

Noticeably, the considered device exhibits an extremely linear behavior during both the set and reset operations, especially if compared with the characteristics of the one-layer device (Figure 7 and Figure 8). However, the pulse voltage required for the switching to occur is considerably larger, starting at 2.5 V for the set process and requiring up to −4 V for the reset process. All these trends are accurately reproduced by the simulations.

While the two-layer stack structure allows for the fabrication of RRAM devices with an intrinsically linear characteristic, the lack of symmetry still prevents their use for ANN and SNN applications and could be detrimental in NVMs. The symmetry of the electrical characteristic can be pursued by further investigating the novel two-layer stack, using the presented modeling platform to optimize the materials and geometries combination, or by using specifically designed pulse sequences. Interestingly, the latter solution is relatively easy to implement: thanks to the intrinsic linearity of the set and reset characteristics, symmetry can be obtained by using different pulses (i.e., with different amplitudes) in the two operations.

### 3.4. Switching Reliability Optimization

The fundamental mechanism enabling analog resistance switching in RRAM devices is the diffusion of oxygen ions in the oxide layer. This process is greatly affected by the interaction of ions with the surrounding lattice, thus by the material’s properties, and in turns affects many of the device’s electrical properties. For example, as previously discussed, precise control of oxygen ion diffusion is key to achieving well-separated resistance levels. Even more importantly, the properties of the ion diffusion process are of paramount importance for evaluating the variability and endurance of RRAM devices [83]. In fact, precise switching between different resistance levels requires the dielectric barrier to be consistently modulated for the full lifespan of the device, with little to no variations between the cycles. Conversely, the newly proposed stochastic learning algorithms for SNNs can take advantage of device variability and non-uniformity. In both cases, the oxygen ion diffusion process must be thoroughly investigated for specific optimization of the devices. The presented multiscale modeling platform, fully accounting for the oxygen ion kinetics, can be used to investigate its effects on the reliability and variability of RRAMs.

**Figure 10:**
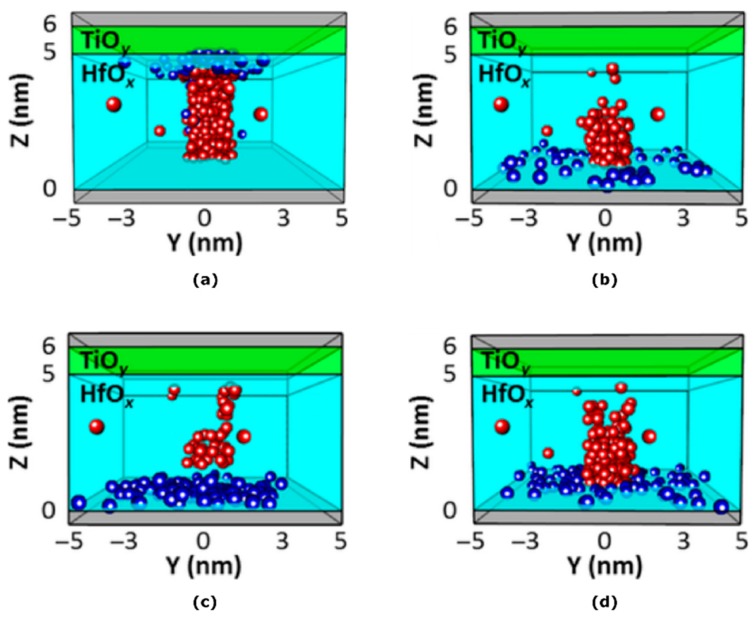
Simulated distributions of oxygen ions (blue) and vacancies (red) in TiN/5 nm HfO_x_/TiO_y_/TiN RRAM stacks: (**a**) after forming; (**b**) after the reset operation with slow and anisotropic oxygen diffusion, showing an efficient reset process; (**c**) after the reset operation with excessively fast and anisotropic oxygen diffusion, showing an inefficient reset process with the formation of a dielectric barrier near the bottom electrode; (**d**) after the reset operation with slow and isotropic oxygen diffusion (with significant motion in the radial direction), showing an inefficient reset process due to the excessive motion of the oxygen ions away from the CF.

The simulations of a TiN/5 nm HfO_x_/TiO_y_/TiN stack RRAM device, shown in Figure 10, reveal the high sensitivity of the device reset operation to the oxygen ion diffusion kinetics properties. Starting from a correctly formed device (Figure 10a), the reset operation was simulated considering slow and anisotropic (motion predominantly in the vertical direction) oxygen diffusion (Figure 10b), corresponding to well-performed reset operation. Then, starting from the same post-formed conditions, the simulation was performed for fast and anisotropic oxygen diffusion (Figure 10c), corresponding to an excessively high voltage reset process, and for slow and isotropic oxygen diffusion (Figure 10d). Interestingly, this last condition describes the diffusive motion of oxygen ions due to the sole temperature field.

An efficient reset operation was only obtained in the first case, where the oxygen ions are carried towards the CF in a mostly anisotropic way (some radial motion is beneficial) and are provided with enough time for recombination with the CF oxygen vacancies. In the case of fast diffusion of the oxygen ions (i.e., for an excessively high-voltage reset process), the latter condition is hindered: a significant portion of the oxygen ions are swept through the oxide, recombining along the CF length and eventually forming a dielectric barrier near the bottom electrode, as suggested in [52]. Finally, the slow and isotropic diffusion of oxygen ions leads to an inefficient reset of the device, as the excessive scattering of ions in the device volume prevents the formation of a fully dielectric barrier. The simulation results are in accordance with the experimental results showing thermally-driven oxygen ions diffusion from the “reservoir” to the CF [84].

The set operation is also affected by the diffusion of newly generated oxygen ions towards the TiO_x_ “reservoir” at the top electrode. Significant radial motion of the oxygen ions during the set operation would spread the ions in the “reservoir”, not creating ion storage in sole proximity to the CF, leading to an increasingly ineffective reset operation and possibly to failure of the device. The cycling endurance of a device is clearly negatively affected by excessive radial motion of the oxygen ions induced by the diffusion. Therefore, convenient process recipes to optimize material properties can be implemented based on the results of these simulations to optimize the endurance and switching uniformity of RRAM devices, depending on the specific application target and its requirements.

## 4. Discussion

Memristors have an extremely high potential for revolutionizing both the memory and AI fields. In fact, memristors have been proposed for implementing high-density, high-speed, and low-power NVMs, and crossbar memory arrays supporting fast and efficient in-memory vector-matrix multiplication for DNN acceleration, and are finding applications in the development of SNNs, enabling the realization of artificial synapses exhibiting an STDP learning mechanism and biologically plausible artificial neurons (i.e., in accordance with the Hodgkin–Huxley model).

As illustrated in Table 1, each of the memristor applications poses different constraints for the performance metrics, demanding devices with rather different characteristics. However, the design and optimization of memristors is still difficult and, most of the time, impractical, due to the limited knowledge on the resistance switching phenomena, their interplay, and the effects of the device materials and geometry on such phenomena.

In this context, the described physical multiscale modeling and simulation provide an extremely powerful tool for the application-oriented optimization of RRAM-based memristors.

As shown in Section 3, the presented multiscale modeling platform allows the device properties, such as its macroscopic electrical properties, its resistance switching dynamics, and its microscopic properties (i.e., the oxygen ions and vacancies distribution), to be simultaneously extracted, and how such properties are affected by the device materials, geometry, and forming conditions to be investigated. Despite a few differences between the simulations and the experimental results, mainly evidenced in Figure 4 and Figure 6, the platform proved capable of capturing the trends and fundamental relationships between the OxRAM devices’ microscopic properties and their behavior.

The discrepancies shown by the simulations can be ascribed to several effects not considered by the platform, such as the oxide thickness and area variations due to the fabrication process tolerances, and process-dependent interface effects between the oxide and the electrodes. The platform can therefore be further improved by including the said effects. Moreover, it could be extended for simulating other memory devices, such as CBRAMs (i.e., including the chemical reaction phenomena at the device interfaces) and Phase Change Memories (PCMs) (i.e., accounting for the phase change in sub-regions of the device).

Nevertheless, the information obtained through the presented multiscale simulation can be effectively used to determine the best combination of OxRAM materials, geometry, forming conditions, and pulse schemes for the desired application, dramatically reducing the time required for its marked deployment.

The application of memristors to NVMs is less demanding performance-wise [26]. Noticeably, for binary NVM applications, the conductance update linearity and symmetry are not required, greatly relaxing both the device and pulse scheme design.

Even in this simple case, device optimization must account for its microscopic properties in order to satisfy the state retention and endurance constraints. In fact, as highlighted by the simulations presented in Section 3.4, the motion of oxygen ions in the device is of paramount importance for achieving an efficient and enduring resistance switching mechanism.

The multiscale simulations can effectively support the optimization of an RRAM-based memristor for NVMs. The device materials, geometry, forming condition, and pulse scheme can be optimized according to the energy consumption and switching time constraints, and the oxygen ions and vacancies distribution can be extracted at the same time. This information can then be used to discard the configurations exhibiting an instable conductive path, thus not satisfying the state retention and endurance constraints.

From the perspective of extending the number of levels of the memristive NVMs, the support of multiscale simulations is even more advantageous, as the higher number of levels requires a tighter resistance distribution of each level. As shown in Section 3.1 and Section 3.2, the resistance variability of the devices can be precisely controlled by finely tuning the forming conditions (i.e., temperature, compliance current, and voltage stress), and the presented simulations allow the best forming condition to be explored.

It should be noted that a device with non-linear and/or non-symmetric resistance switching can still be used in multilevel applications if compensated for by designing a suitable pulse scheme [85,86,87], as shown in Section 3.3. With the support of the described multiscale simulations, the required pulse scheme can be co-designed with the device properties and optimized to achieve the required number of levels. However, linear and symmetric resistance switching would be highly desirable, as it allows for easier and more effective level control, without requiring complex pulse schemes.

As shown in Section 3.3, using a two-layer geometry RRAM allows linear resistance switching to be achieved. In this case, the interplay between the resistance switching phenomena of the two different dielectrics determines an extremely complex behavior, which can be effectively investigated through a multiscale simulation of the device. With the support of the extracted information, the two-layer structure (i.e., its materials and geometry) can be easily optimized to achieve the desired linear, and possibly symmetric, resistance switching.

The implementation of a DNN accelerator with a crossbar memory array architecture requires memristors with very different characteristics [39].

First, the number of levels is dictated by the weight precision required by the implemented DNN: a higher number of levels corresponds to a better learning capability, higher inference accuracy, and higher robustness. A precision as low as 6 bits (i.e., 64 levels) has been shown to be effective for both training and inference [88]. Moreover, the resistance switching must be absolutely linear and symmetric to avoid significant losses in the DNN accuracy [89]. This is especially true for online-trained DNNs, while for offline-trained DNNs, the non-ideal conductance update can be compensated for by suitable writing schemes.

For this application, a two-layer structure is clearly beneficial and, as stated before, the support of the described multiscale simulations allows a better understanding of the device’s complex behavior and optimization of its structure to match the requirements.

Due to the high number of levels required, the dynamic range must increase in parallel with the weight precision to ensure a sufficient separation of the levels, while ensuring a sufficiently high resistance value of the low-resistance state in order to limit the inference energy consumption. The energy consumption of the training phase is instead determined by the programming energy, which must be limited. A related performance metric is the switching time, which must also be as low as possible to ensure both fast and energy-efficient training.

All of these metrics are interconnected and their relationship with the device geometry and materials, and their trade-offs cannot be easily appreciated and designed. The low-resistance state value and the variability of the resistance levels can be controlled by the forming conditions, as previously stated and as shown in Section 3.1 and Section 3.2, but the effects on the required programming energy and switching time are not obvious. The multiscale simulation provides an extremely powerful tool to explore the possible solutions and possibly optimize the device materials, geometry, and forming conditions to match the requirements.

Finally, the reliability-related metrics (i.e., endurance and retention) are extremely demanding and critical: the training phase (or the weights set up in the case of offline training), requiring a large number of switching operations, can be stressful for the devices and the weight values must be retained (ideally) indefinitely. As for the NVM application, the stability of the conductive path in a specific device can be easily investigated though multiscale simulations, allowing for the recognition of unsuitable solutions.

The desired performance for memristors implementing SNN artificial synapses is extremely similar to that required for DNN accelerators [40], especially those concerning the number of levels and the reliability-related metrics (i.e., endurance and retention). This is reasonable, since SNNs also undergo a stressful training phase (or synaptic weight set up), requiring many switching operations. Moreover, the specific requirements for feature size and switching time can be reasonably assumed to be similar to those of DNN accelerators. Additionally, recent works suggest that artificial synapses with symmetric conductance updates allow for a better accuracy in SNNs [90].

Noticeably, the application-level effects of memristors’ stochasticity, i.e., uniformity (or lack of thereof) and variability, are little discussed in the literature. In NVM applications, both the non-uniformity and high variability of the memory cell can be detrimental [39], posing a challenge for technology development. Neural network applications exhibit a good tolerance to device-to-device and cycle-to-cycle variations, especially if online training is used [88,89]. Interestingly, the exploitation of memristors’ stochasticity has recently been proposed for implementing stochastic learning algorithms for SNNs [22,91]. In such a context, the device’s variability, non-uniformity, and noise become key components of the learning algorithm, thus requiring a precise design. All those properties can also be investigated using multiscale simulations, e.g., as previously stated, the device’s variability can be controlled and optimized by tuning the forming conditions.

## 5. Conclusions

We have shown that multiscale modeling and simulation can effectively support the application-oriented optimization of RRAM devices.

With the support of the presented multiscale modeling platform, we simulated the microscopic behavior of RRAMs and investigated the effects of the device geometry, materials, forming conditions (i.e., temperature, current compliance, voltage stress mode), and programming on the device performance. The multiscale simulations allowed the properties of RRAMs during their whole operation, from the forming process to the subsequent set-reset cycle, to be investigated, providing information about the device linearity, symmetry, dynamic range, and reliability.

The presented multiscale simulations provide useful design principles for RRAM technology optimization according to the specific AI application, for the implementation of non-volatile memories, deep neural networks, or spiking neural networks.

Moreover, the multiscale simulation allows the effects of different implementations of the device (i.e., different geometries or materials) to be explored, and both the forming conditions and the pulse scheme (i.e., amplitude, width, sequence) to be finely tuned for achieving the desired performance.

## Figures and Tables

**Table 1:** Summary of desired performance metrics for memristors.

Metric	NVM ^1^ [26]	DNN ^2^ [39]	SNN ^3^ [40]
Feature size	<12 nm	<10 nm	-
Number of levels	≥2 (1 bit)	>100 (6.45 bits)	≥64 (6 bits)
Dynamic range (on/off ratio)	-	≥100	≥00
State retention	>1 year	>10 years	>10 years
Device endurance	>10^3^ cycles	>10^9^ cycles	>10^9^ cycles
Energy consumption	<100 pJ/write	<10 fJ/programming pulse	<10 fJ/spike
Linearity	-	Yes	Yes
Symmetry	-	Yes	-
Switching time	<100 µS	<100 ns	-

^1^ System level performance for replacing a NAND flash memory. ^2^ Performance for memristors in a deep neural network (DNN) accelerator with a crossbar memory array architecture. ^3^ Performance for memristors as a Spiking Neural Network (SNN) artificial synapse.

**Table 2:** Simulation parameters.

Symbol	Quantity	Material		
		HfO_2_	Ta_2_O_5_	TiO_2_
**Material Parameters**				
E_G_	Band-gap (eV)	5.8	3.6	1.3
χ	Electron affinity (eV)	2.4	3.4	4.3
k	Relative dielectric permittivity	21	25	95
m_e_*	Electron tunneling effective mass	0.25 m_0_	0.3 m_0_	0.2 m_0_
WF	TiN work function (eV)	4.57	4.57	4.57
**Defect Parameters**				
E_T_	Defect thermal ionization energy (eV)	1.7–2.7	0.8–1.2	0.1–0.5
E_REL_	Defect relaxation energy (eV)	1.19	0.88	0.7
N_T_	Defect density (cm^−3^)	5 × 10^19^	5 × 10^19^	5 × 10^19^
**Metal-Oxygen Bond Breakage Parameters**				
p_0_	Polarizability (eÅ)	5.2	1.8	4
E_A,G_	Activation energy (eV)	2.1	1.0	5.3
G_0,G_	Effective bond vibration frequency (Hz)	4.5 × 10^13^	4.5 × 10^13^	4.5 × 10^13^
**Oxygen Ion Diffusion Parameters**				
Γ	Field acceleration factor (eÅ)	0.3	0.2	0.4
E_A,D_	Activation energy (eV)—in x/y/z direction	0.8/0.8/0.7	1.2/1.2/1.0	1.0/1.0/0.75
G_0,D_	Effective bond vibration frequency (Hz)	4.5 × 10^13^	4.5 × 10^13^	4.5 × 10^13^
